# South West Orthopaedic Club

**Published:** 1981

**Authors:** 


### Abstracts

### South West Orthopaedic Club
2nd May 1981, Truro

### PRIDIE MEMORIAL LECTURE

**Mr. M. A. R. Freeman**
**London Hospital Medical College**

The results were reported of double cup hip replacements carried out at The London Hospital between the 1st January 1975 and the 31st December 1977. The prosthesis used during this period consisted of a cemented metalic femoral component and a cemented, approximately hemispherical, polyethylene acetabular component.

Successfully replaced hips during this period are now available with a follow-up of up to six years. Their symptomatic state is equivalent to that of conventional hip replacement.

Thirty-five percent of hips treated in the period have failed although not all have subsequently required revision surgery. The principle causes of failure were as follows:

1. **Errors of Indication**
   Forty percent of all failures were in patients having a replacement for inflammatory arthritis (rheumatoid arthritis or ankylosing spondylitis) or rapidly progressive osteoarthrosis. It is now felt that the operation should be carried out rarely, if at all, in these conditions.
2. **Femoral Failures**
   These were seen as a consequence of two technical errors: notching of the superior neck (which it is speculated may interfere with the interosseous blood supply to the head and thus lead to femoral loosening) and varus placement of the femoral component (which may lead to subcapital fracture or femoral loosening).
3. **Errors of Acetabular Design and Implantation Technique**
   The fundamental problem in this connection is that the acetabular component used in the period under study was too large and thus tended to protrude from the pelvis. The protruding polyethylene sometimes impinged against the femoral neck, resulting in pain, stiffness and or acetabular loosening. Other factors which it was thought might increase the chances of acetabular loosening and perhaps increase the incidence of significance radiolucent lines around the acetabular cement were: the presence of excess cement in the floor of the acetabulum, the presence of too thin a layer of cement superiorly, the fact that the wall thickness of the polyethylene component is less than that of a conventional prosthesis and finally that the radius of the femoral head is greater than that of a conventional prosthesis.

Some possible solutions to these problems, used clinically in the years 1980/81, were discussed.

### THE DEANE KNEE

**A clinical review of the prosthesis in development**

**Christopher E. Ackroyd, Peter C. Mattingly and Graham Deane, Bristol**

A new concept of a stable surface knee replacement was designed in 1972. Clinical trials were commenced in 1974 at the Nuffield Orthopaedic Centre. Sixty-two total knee joint replacements have now been performed in 54 patients. Twelve patients were suffering from osteoarthritis and the remainder suffered from rheumatoid arthritis. The first 23 patients were nursed post-operatively with the knee in extension for two weeks: four of these patients required manipulation under anaesthesia in the post-operative period. A post-operative knee flexion regime was introduced after Case 24 and no subsequent patient has required manipulation under anaesthesia. There has been a reduction in the incidence of wound healing problems since this regime was introduced. There have been no cases of persistent deep joint infection, although 2 patients developed late infection, one due to a streptococcal cellulitis which was successfully treated by antibiotics and the second due to a traumatic leg ulceration which led to septicaemia and death. Two patients developed a late low grade inflammatory response which has been controlled by antibiotics.

A clinical and radiological review has been carried out by CEA and PCM on the first 55 cases which have been followed for 10–60 months. Six patients have died (seven knees) and 3 patients are unavailable for follow up. Forty-five knees have been examined in 40 patients and the results of this review are presented.

A subjective assessment of the patients' satisfaction has been carried out using a visual analogue scale: 71% were enthusiastic or very satisfied, 7% non-committal and 22% were disappointed. A more objective grading system was designed, based on Insall's scoring system but weighted towards pain and function. 66% of the knees had an excellent or good result. 22% were fair and 11% were poor. The most important late complication was that of loosening of the tibial component which was definitely present in 8 knees and possibly present in 4 knees. Four of these have been revised and 2 are now satisfactory. Four remaining patients have a loose prosthesis, however, two have satisfactory knees and a further two will be revised shortly. There are four further cases which have significant pain which could be due to impingement or patello-femoral changes. 72% of the cases have excellent relief from pain. Of those patients with moderate or severe pain, the cause is loosening in half and other causes are patello-femoral pain, hip pain or tibio-femoral impingement. Analysis of knee movements shows that only two patients have a maximum of less than 80° and four patients have a fixed flexion deformity of 20° or more.

The results of this review have identified one important design error which has led to the main conplication of tibial loosening. As a result, continuing development has led to the design of a definitive tibial component with more secure tibial fixation which has been verified by laboratory evidence. In other respects the prosthesis functions well with good stability and a satisfactory range of movement. There has been no evidence of wear of the high density polyethylene femoral component.

### THE THREE STAGE TREATMENT OF SEVERE OPEN FRACTURES

**D. J. Bracey, Truro**

This staged treatment is useful in dealing with severe shaft fractures of the femur and tibia with extensive soft tissue wounds. The first stage comprises a debridement of the wound and fixation of the fracture, either using an external fixator or plate. In the tibia the plate would be placed on the lateral side under the cover of the anterior muscles such that the wounds can be left open without metal ware exposed. One week later the second stage is performed. This is a further debridement and a corticocancellous bone graft introduced through the open wound which is again left open. The third and final stage is a split skin graft usually applied after a further delay.

The essential features are thus, open wound treatment, rigid fixation of the fracture and prophylactic bone grafting. The results of 38 compound fractures of the tibia and femur treated at Sunnybrook Medical Centre, Toronto were presented. Two patients required immediate below knee amputation for severe tibial fractures leaving 30 patients with 36 fractures for review. All of the fractures united except two which were associated with popliteal artery obstructions. One of these led to a below knee amputation after four days and another resulted in an infected non-union. Of the cases which united two had osteomyelitis, an overall deep infection rate of 5%.

### FEMORAL FRACTURES IN RELATION TO CEMENTED HIP ENDOPROSTHESES

**P. H. Cooke, J. H. Newman, Bristol**

Femoral fractures occurring in the presence of hip prostheses are well recognised and appear to be an increasingly common clinical problem. Although much has been written about those fractures occurring during operation, the experience of post-operative fractures is largely limited to those around uncemented prostheses.

Twenty-nine femoral fractures occurring after operation in association with cemented hip endoprostheses were studied. The majority occurred at the tip of the prosthesis (Type III), though fractures around the stem (Type II) and distal to the prosthesis (Type IV) also occurred. Four fractures were through known cortical defects, and these all occurred within one year. However, the remaining 25 occurred on average 7.7 years after operation and the possibility of femoral shaft weakening due to the presence of a rigid prosthesis is raised.

Non-operative management led to loosening of the femoral component following Type II fractures, and mal-union or non-union is all Type III fractures. Operative results were marred by infection, bending and malposition of the prosthesis. However, in the light of modern knowledge, many of these problems are avoidable, and operative management of all except Type IV fractures is therefore advocated.

### NEGLECTED UNUNITED FRACTURES OF THE INTERCONDYLAR AREA OF THE TIBIA

**M. Halawa, Exeter**

Fractures of the intercondylar area of the tibia are known in the orthopædic literature as fractures of the tibial spine. They are of two types:
(i) Anterior. This type bears the attachment of the anterior cruciate ligament.
(ii) Posterior type bears the attachment of the posterior cruciate ligament.

Fresh fractures of the above types ought to be reduced anatomically and better to be internally fixed.

Failure to do so will result in non-union which would cause block to full extension in the anterior type and giving way in the posterior type.

This Paper deals with the treatment of ununited fractures of the intercondylar area of the tibia and its problems. Four cases of the anterior type were dealt with. All presented with 30–40° block to full extension.

Three were treated by open reduction, two with internal fixation and one just forced hyperextension was applied to maintain the reduced position. Above knee plaster in full extension of the knee was applied in all for six weeks. Satisfactory result was generally obtained in all of them after a period of rehabilitation. One case obtained in all of them after a period of rehabilitation. One case was treated by exision of the ununited fragment. The resultant antero-lateral instability was treated by vigorous quadriceps exercises.

Two cases of the posterior type were treated by open reduction and internal fixation. The posterior instability of the knee and the posterior draw sign were improved, but normality was not achieved by comparison with the other knee.

The results of open reduction and internal fixation of the posterior type fractures are not as good as the result of the open reduction and internal fixation of the anterior type. This may be mainly because of accompanying old posterior capsular tear.

### TENNIS ELBOW – GARDEN v MAUDSLEY

**G. Heyse-Moore, Exeter**

The results of surgical treatment of persistent tennis elbow carried out by one orthopædic surgeon in Truro over 20 years were reviewed.

There were operations of 51 elbows, consisting of 36 lengthenings of the extensor carpi radialis brevis tendon and 15 neurolyses of the posterior interosseous nerve under the arcade of Frohse. Pre-operative symptoms and signs were the same in all patients except three with weakness of wrist extension, thus apart from these three who probably had a true radial tunnel syndrome it was felt that the same complaint was being treated by two different operations.

The results of operation were 89% success for the ECRB legthening and 80% success for radial nerve neurolysis, thus was not significantly different.

It was shown that broad and tender scar was a significant complication of posterior interosseous nerve decompression.

A cadaver dissection was illustrated to demonstrate that the operation of posterior interosseous nerve neurolysis could work by releasing tension on the common extensor origin by dividing the superficial leaf of supinator, this mechanism being the same as lengthening the extensor carpi brevis tendon and threrfore not necessarily due to decompressing the posterior interosseous nerve.

It was concluded that of the two operations the Garden procedure was the one of choice.

### COMPARISON OF MENISCECTOMY BY 'OPEN' AND 'CLOSED' TECHNIQUES

**P. K. Peace, Truro**

The importance of partial rather than total meniscectomy was stressed and reference made to the pioneering work of Jackson in Toronto who first performed a partial meniscectomy by the 'closed' technique in 1970 and more recently by Dandy in Newmarket.

A small series was submitted following results of questionnaires sent to 57 patients. 46 replies were received but 14 of these were excluded because of severe osteo-arthrosis or instability of the knee. 16 patients were reviewed in each group. The 'open' group having 14 males and 2 females with an average age of 38.3 years and the 'closed' group having 15 males and 1 female with an average of 34.7 years.

The operative details in the 'open' group were that all cases were subjected to preliminary arthroscopy, tourniquet then inflated after elevation of the leg and the knee was re-towelled and the surgeon regowned. A 3" oblique incision used and plaster immobilization for 10–14 days post-operatively.

The 'closed' technique was performed with a tournique inflated from the outset and 6mm incision on each side of the knee and a pressure bandage applied post-operatively.

In the 'open' group nearly all cases were involving the medial meniscus and in the 'closed' group there was a slight preponderance of lateral menisci involved. There were relatively few bucket handle tears in the 'closed' group mainly due to lack of experience during the period of review.

The results showed the number of days in hospital for the 'open' group 4.3 days and the 'closed' group 2.2 days. Physiotherapy ofter operation was required by all but one in the 'open' group for an average of 4 weeks and by only 3 in the 'closed' group. The number of weeks off work following meniscectomy in the 'open' group was 5.4 weeks and in the 'closed' group 3.2 weeks. The relationship to sports of the two groups showed that 9 in each group stated that the cause of their knee injury was through sport. After operation only 5 out of 11 declared sportsmen in the 'open' group returned to sport but 12 out of 13 returned to sport in the 'closed' group.

The mean follow-up in the 'open' group was 16.7 months and in the 'closed' group 5.5 months. From the questionnaire current symptoms were assessed and in the 'open' group 7 had none, 8 had 'minor' symptoms and only one had significant symptoms. In the 'closed' group 6 had non, 7 had 'minor' and 2 had significant symptoms. Unsolicited favourable comments were received from 4 patients in the 'open' group and 7 patients in the 'closed' group.

Conclusions are that 'closed' partial meniscectomy gives rise to a shorter hospital stay, and earlier return to work and sports. The long-term results are awaited with interest.

### FEMORAL FRACTURE CAST BRACING IN EXETER

**Ian B. M. Stephen, Exeter**

This paper describes the evolution of the system of cast bracing for femoral shaft fractures currently sued in Exeter. Various modifications of the conventional cast brace have been introduced to overcome its disadvantages, and incorporated in the system during a prospective study over three years.

Thirty four patients were treated by early bracing at an average time of six weeks after fracture; thirty united without problems, although fractures of the upper shaft tended to unite in varus and flexion. Eight patients were treated by lat bracing at an average of 14 weeks after fracture and six united without problems. The commonest reason for discontinuation of bracing was skin maceration, especially when polythene components were used.

The system currently used is to reduce the fracture primarily by manipulation and maintain skeletal traction until the fracture is 'sticky'. A cast brace is then applied, on the hip table, over a tubigrip stocking. Well moulded gypsona is used for the thigh piece, connected to a plastic below knee cosmetic caliper with partly constrained metal coil twist-brace hinges. Radiological control is used to confirm a satisfactory position. Weight bearing and knee flexion are commenced in 24 hours, and the brace is maintained until union is evident radiologically.

This system of treatment for femoral shaft fractures has been found to be effective and inexpensive, as well as having significant advantages over other methods.

